# Molecular detection of black queen cell virus and Kashmir bee virus in honey

**DOI:** 10.1186/s13568-018-0655-7

**Published:** 2018-08-07

**Authors:** Vesna Milićević, Sonja Radojičić, Jasna Kureljušić, Milanko Šekler, Ksenija Nešić, Ljubiša Veljović, Jelena Maksimović Zorić, Vladimir Radosavljević

**Affiliations:** 1 Virology Department, Institute of Veterinary Medicine of Serbia, Vojvode Toze 14, Belgrade, 11000 Serbia; 2 Infectious Animals Diseases and Diseases of Bees, Faculty of Veterinary Medicine, Bulevar Oslobodjenja 18, Belgrade, 11000 Serbia; 3 Institute of Veterinary Medicine of Serbia, Vojvode Toze 14, Belgrade, 11000 Serbia; 4 Veterinary Institute “Kraljevo”, Žička 34, Kraljevo, 36000 Serbia

**Keywords:** Honey, RT-PCR, Honey bee viruses, Phylogenetic analysis

## Abstract

Considering the intensive trading nowadays, the honey from the local market was tested for the presence of the six most common bee viruses. To prove the suitability of honey as a sample for the bee viruses detection, the set of different sample types taken directly from the hives we comparatively tested. The study included 30 samples of domestic and 5 samples of imported honey. Additionally, we tested 40 sets of samples including live bees, dead bees, and the honey taken from four apiaries for the evaluation of honey suitability for the virus detection, Two out of the six most common bee viruses were detected in the samples of honey from the market. Black queen cell virus (BQCV) genome was found in 24 domestic honey samples and Kashmir bee virus (KBV) genome was detected in one sample of imported honey. The nucleotide sequences of 24 BQCV isolates showed the highest identity (86.4%) with strains from Europe at the polyprotein gene, whilst the Serbian isolates between each other showed 98.5% similarity. By comparative testing of the different type of samples, in three out of four apiaries BQCV genome was detected in both bees and honey. Evaluating the suitability of honey for the detection of the viral disease by simultaneous testing of live, dead bees, and honey from the same hive, it was shown that the honey can be successfully used for the detection of BQCV. Since, as of yet, there has been no evidence of KBV circulation in Serbia, after its detection in imported honey, there is a substantial risk of its introduction and consequently the need for its surveillance. Therefore, the programs of bee diseases screening should be included in the regular control procedures for the international trade. In addition to this benefit, honey gives an opportunity to beekeepers for continuous monitoring of bees’ health status.

## Introduction

Traditionally honey and other honeybee products are considered as a healing food. International standards for the quality of honey, specified in European Honey Directive and in the Codex Alimentarius Standard for Honey, oblige regular laboratory control of essential composition and quality factors (moisture content, sugars content, water-insoluble solids content), contaminants (heavy metals, residues of pesticides and veterinary drugs) and hygiene. However, this investigation does not include tests for infectious diseases. Despite many preventive measures, irresponsible human activities can lead to disease spreading into new regions and continents, including non-zoonotic diseases. As an example, though Colony Collapse Disorder is not primarily an infectious disease, it can be considered as a “human-borne” disease (Pettis et al. 2013).

Nevertheless, many bee viruses are very common and widespread. In general, bee viral infections are asymptomatic, often recognized only as a shortened lifespan, but under certain conditions, the viruses can cause severe and often fatal consequences (Berényi et al. 2006).

There are currently about 24 viruses (Gisder and Genersch 2015) identified in honey bees, but acute bee paralysis virus (ABPV), black queen cell virus (BQCV), deformed wing virus (DWV), Sacbrood virus (SBV), chronic bee paralysis virus (CBPV) and Kashmir bee virus (KBV) are, with a worldwide distribution (Allen and Ball 1995), considered to be able to cause severe disease in honeybees.

Diagnosis of honeybee viral infections commonly relies on clinical symptoms, which, often, are not clearly manifested or are manifested only during the particular life stages. It is also known that different viruses can produce similar symptoms (e.g. paralysis) (Gupta and Reybroeck 2014). The symptom-based diagnosis is robust, simple, fast and cheap and for some diseases accurate, but it is often too late for proper action or not reliable (Gupta and Reybroeck 2014). Nowadays, for routine laboratory diagnosis, polymerase chain reaction (PCR) is the most widely used method providing the virus detection even if the virus is not viable or the ribonucleic acid (RNA) has been degraded by endogenous ribonucleases. Many protocols for either individual or multiplex detection of specific honeybee viruses have been described whereas live or dead bees are considered as the most reliable and suitable samples for laboratory investigations (Chen et al. 2004). However, honey is commonly used for some bacterial diseases detection such as American foulbrood (Lauro et al. 2003),

Recent losses of honeybee colonies at the global level have led to the increased interest and intensive investigation of any threats to honeybee health in our country. Therefore, the objective of this study was to investigate the presence, to characterize the bee viruses in the honey from the market, and to assess the risk it carries. As there are no studies, according to our knowledge, which uses honey as the sample for viral diseases detection, to prove the suitability of honey for this purpose, the set of different samples from the hives were comparatively tested. Additionally, the aim of this study was to recommend the hive honey testing for routine health control of bees, since beekeepers are not always capable to recognize pathological processes in hives, particularly during the asymptomatic phases.

## Materials and methods

### Samples

For simultaneous PCR detection of six major bee viruses, 35 honey samples, packed and stored in 1 kg glass jars (30 samples originating from 12 different regions in central and northern Serbia and 5 imported), from the local markets were used. The honey was sampled during the 2015 and 2016.

Also, for the evaluation of honey suitability for detection of bee viral diseases, samples of both honey, and live and dead bees were collected at the same time from same hives. In total, 40 samples of bees and honey from four apiaries (15 samples from apiary I, 5 from apiary II, 13 samples from apiary III and 7 samples from apiary IV) were examined. The apiaries, in a range of 10 km radius, were located in central Serbia region and had no visible disorders.

### RNA isolation and RT-PCR

To detect the viral genome of BQCV, KBV, DWV, ABPV, SBV, and CBPV, we used conventional, multiplex RT-PCR. Prior to RT-PCR, viral RNA was isolated using commercial Isolate RNA Mini Kit, Bioline, UK.

As, according to our knowledge, there is no available protocol for RNA isolation from honey, we have prepared the samples as 1:2, 1:5, 1:10 and 1:100 dilutions of honey in PBS and applied the protocol for RNA isolation from cell cultures. Each dilution was used as a starting material for RNA extraction.

From each hive, 10 bees were homogenized by mortar and pestle in 5 ml sterile phosphate-buffered saline (PBS). After homogenization, the samples were centrifuged for 10 min at 2000 rpm. The supernatant was used for RNA extraction.

RT-PCR was carried out using Verso 1-Step RT-PCR kit ReddyMix (Thermo Scientific). Reactions of 25 µl were composed of 5 µl template RNA, 12.5 µl 2 × 1-Step PCR ReddyMix, 1.25 µl RT Enhancer, 0.5 µl Verso Enzyme Mix and 0.5 µl of each of 12 primers (10 µM) previously published (Stoltz et al. 1995; Benjeddou et al. 2001; Ribiere et al. 2002; Tentcheva et al. 2004, 2005).

Amplifications were accomplished throughout incubation step at 50 °C for 30 min, 40 cycles of incubations at 94 °C for 30 s, 55 °C for 1 min and 72 °C for 2 min, and single step of final extension at 72 °C for 7 min.

The amplified products were electrophoresed on 2% agarose gel containing ethidium bromide, and visualized by UV transillumination. Thermo Scientific Gene Ruler 100 bp was used for sizing of PCR products on the gel.

### Nucleotide sequencing and computer analysis

The specific amplicons were sequenced in Macrogen Europe, The Netherlands. The sequences were compiled and aligned using MUSCLE. The phylogenetic tree was constructed in MEGA6 software, using the Maximum Parsimony method and applying bootstrap resampling of 1000 replicates, in order to prove the stability of the trees. Tajima Nei model was used to compute the distance matrices.

## Results

In examined honey samples from the market, the presence of two (BQCV and KBV) out of the six examined bee viruses was detected. All 25 samples tested positive at the dilutions from 1:2 to 1:10, but only 21 were positive at 1:100 dilution of the honey sample.

The BQCV genome was found in 24 honey samples (80%) originating from Serbia. KBV genome was detected in one sample (20%) of imported honey. The BQCV sequences were submitted to GenBank database under accession numbers KX591576–KX591599. However, KBV was neither investigated nor sequenced.

**Fig. 1:**
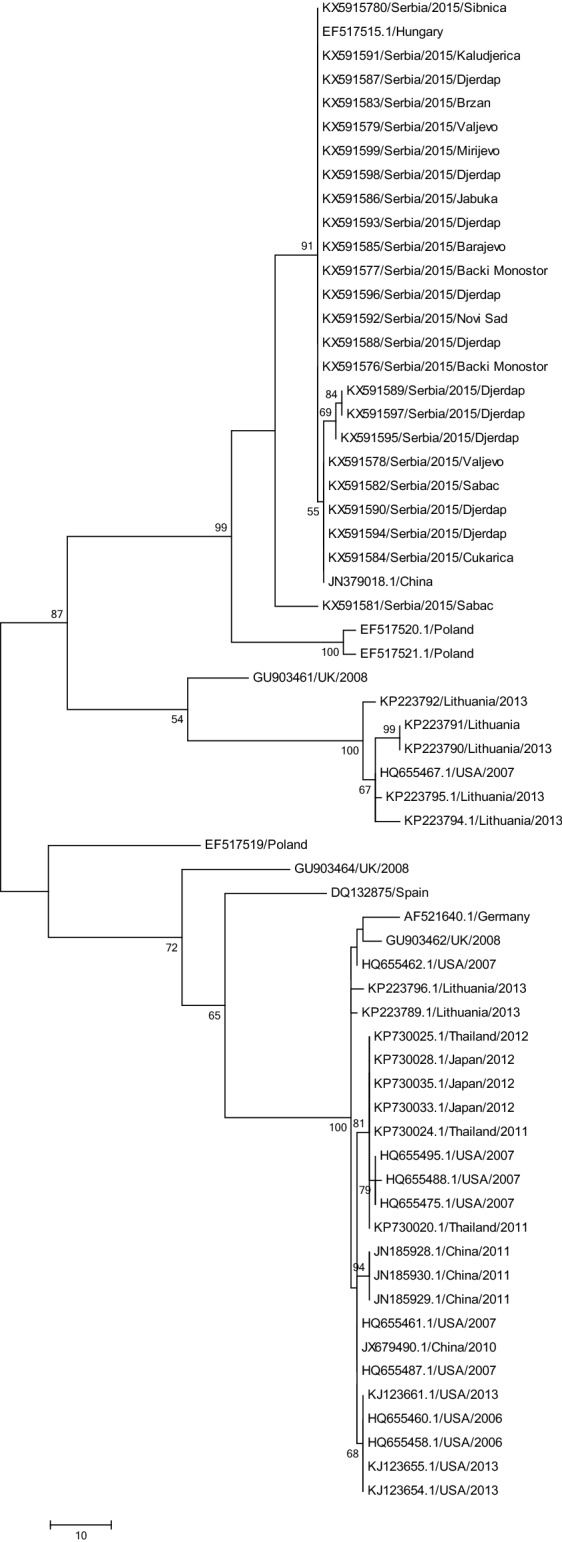
Phylogram illustrating the genetic relationships among BQCV isolates, based on the partial RNA polyprotein coding region. Evolutionary analyses were conducted in MEGA6 using maximum-parsimony. The analysis involved 63 nucleotide sequences. The percentage of replicate trees in which the associated taxa clustered together in the bootstrap test (1000 replicates) are shown next to the branches; only values > 50% were indicated. Scale bars indicate the numbers of steps for parsimony analysis

The phylogenetic tree (Fig. 1) based on the partial polyprotein coding region of BQCV is divided into two main groups. The first group consists of isolates from USA and Asia. The other group contains European BQCV isolates including the 24 isolates from Serbia. Two branches are distinguished in the latter group, with Lithuanian isolates and one isolate from the UK in the first branch. The second one is formed out of two separate interior branches, one of which is made of two Polish isolates. Serbian isolates formed two lineages: the first one that is the closest to the Hungarian isolate and includes a sublineage clustered together with one Chinese isolate, and the second one formed from one Serbian isolate from Sabac region. At polyprotein domain, Serbian BQCV isolates showed the highest identity (86.4%) with strains from Europe, whilst the most divergent strains were from the USA. Serbian isolates between each other showed 98.5% similarity.

BQCV was the only virus found in the bee and honey samples from apiaries. The virus was detected in three out of four apiaries. In apiaries I, III and IV BQCV genome was detected in both bees and honey (Table 1). BQCV genome was not detected in either honey or bees from the apiary II. Black queen cell virus prevalence at a hive level was between 86.6 and 100% (Table 1).

**Table 1:** Results of molecular tests on bees and honey to demonstrate suitability of honey as sample for viral diseases detection

Apiary	Bees		Honey	
	Positive hives	Negative hives	Positive hives	Negative hives
	No (%)	No (%)	No (%)	No (%)
I	13 (86.7)	2 (13.3)	13 (86.7)	2 (13.3)
II	0	5 (100)	0	5 (100)
III	12 (92.3)	1 (7.7)	12 (92.3)	1 (7.7)
IV	7 (100)	0	7 (100)	0

## Discussion

In our research, we showed that BQCV is widely present in domestic honey available from the local markets, while KBV was only found in imported honey. In order to avoid difficulties in RNA extraction due to the viscosity and inhibition in subsequent RT-PCR, serial dilutions of honey in PBS were prepared and tested. Detection of those viruses in 10^2^ dilution in 84% of the samples led to the conclusion that infections of bees with either BQCV or KBV consequently result in a high viral titer in honey. We’ve presumed that the initial lower viral load was the crucial reason for its limited detection at higher dilutions (10^2^) in four samples rather than the RNA degradation that might have occurred during the transport and storage.

As opposed to this finding, a study by Dainat et al. (2011) showed that if bees were used, even stored at + 4 °C, the RNA was partially degraded within 1–3 days, with total RNA degradation further after 5 days. However, the authors found faster degradation of cellular RNA than degradation of viral RNA in bee samples.

Having found BQCV in the high prevalence in honey from the market whereas KBV was only found in an imported one, the concern on whether those results reflect the real situation in the field has been raised. Black queen cell virus (BQCV) has been reported in America, Europe, Asia, Africa and the Middle East (Ellis and Munn 2005). According to Petrovic et al. (2013), the most prevalent honey bee virus in Serbia is BQCV. In neighboring Croatia, 40.24% of apiaries were infected with BQCV (Gajger et al. 2014). BQCV was identified in 21.42% apiaries in Turkey (Gumusova et al. 2010), 30% in Austria (Berényi et al. 2006), 86% in France (Tentcheva et al. 2004).

However, Kashmir bee virus, the most virulent of all known honeybee viruses (Allen and Ball 1995), has not been reported in Serbia so far. Kashmir bee virus is endemic in Australia and in the United States (Berényi et al. 2006). In Europe, KBV has been found in Spain, France, Germany, Luxemburg, the United Kingdom and Denmark (Nielsen et al. 2008). Like most dicistroviruses (de Miranda et al. 2010), KBV persists at low titers in apparently healthy colonies until several stress factors activate the viral multiplication causing the death of the colony.

With regard to these reports, our results obtained from market honey could be considered as representative for the bee viruses presence at the country level rather than at apiary level, since the honey in the market is usually a mixture of the honey of different origin.

Further on, to prove the suitability of honey as a sample for viral diseases detection, we compared the results obtained from testing both honey and bees from the same hives. Despite no observed clinical signs, BQCV was found in the highest percentage. The observed correlation between results obtained from honey and bees was very high as BQCV was simultaneously detected in honey and bees from the same hive with no observed deviation. Thus, BQCV could be discovered, by testing honey instead of healthy bees, even before the occurrence of any visible clinical sign. As BQCV is usually controllable in most colonies, the beekeeper could timely, before overwintering, take actions to prevent the outbreak (Gupta and Reybroeck 2014).

Unfortunately, since the other viruses were not detected in hive samples, the usefulness of honey for the detection of other bee viruses needs to be additionally confirmed.

Besides significance in global epidemiology and prevention of fatal diseases, the investigations of invertebrate viruses may also provide information on differences in the pathophysiology, immunology, and ecology of invertebrate and vertebrate infections (Tapaszti et al. 2009). Furthermore, bees can be used as a model for the spread of disease in the light of intensive trading nowadays.

The phylogenetic analysis for the polyprotein genome region of the Serbian BQCV strains detected in market honey showed that all Serbian isolates were clustered together, similarly to the other strains from different countries, which were clustered by geographical origin. However, one Serbian isolate formed a separate branch, indicating the different origin of the virus. The low bootstrap value, however, indicates the slow evolution.

The high similarity between Serbian and Hungarian isolates was expected, taking into consideration the vicinity of the countries, habitat, and pastures. Unexpectedly but quite strongly supported, phylogenetic analysis revealed a Chinese strain clustering with Serbian isolates. However, according to the Chamber of Commerce and Industry of Serbia, there has been neither honey nor bee products from China on Serbian market. To determine the origin of this strain, for further investigation, full genome sequences are needed.

Although no infectivity test was performed, the import of honey should be treated as a risk for the viral introduction into free areas. Though it is unlikely that feeding would be the route of transmission, the leaks in shipping containers could lead to ‘robbing’ of the honey by foraging bees and discarded honey containers may also be accessible to honey bees (Shimanuki and Knox 1997).

To date, there has been no evidence of KBV circulation in Serbia. Nevertheless, after KBV detection in imported honey, there is a substantial risk of its introduction and therefore consequently the need for its surveillance. Therefore, the programs of bee diseases screening should be included in regular control procedures for the international trade. In addition to this benefit, honey gives an opportunity to beekeepers for continuous monitoring of bees’ health status. By testing honey, owners would be able to timely react and prevent massive losses.

## Abbreviations

ABPV: acute bee paralysis virus
BQCV: black queen cell virus
CBPV: chronic bee paralysis virus
DWV: deformed wing virus
EU: European Union
KBV: Kashmir bee virus
PBS: phosphate-buffered saline
PCR: polymerase chain reaction
RNA: ribonucleic acid
RT-PCR: reverse transcription-polymerase chain reaction
SBV: Sacbrood virus
